# Establishment and validation of a novel prognostic model for non-virus-related hepatocellular carcinoma

**DOI:** 10.1186/s12935-022-02725-5

**Published:** 2022-10-02

**Authors:** Yu Jiang, Shulin Chen, Yaxian Wu, Yuanye Qu, Lina Jia, Qingxia Xu, Shuqin Dai, Ning Xue

**Affiliations:** 1 Department of Clinical Laboratory, Affiliated Tumor Hospital of Zhengzhou University, Henan Cancer Hospital, Zhengzhou Key Laboratory of Digestive System Tumor Marker Diagnosis, 127 Dongming Road, Zhengzhou, 450000 China; 2 Department of Clinical Laboratory, State Key Laboratory of Oncology in South China, Collaborative Innovation Center for Cancer Medicine, Sun Yat-Sen University Cancer Center, Guangzhou, 510060 China

**Keywords:** NV-HCC, Prognosis model, LASSO-Cox regression

## Abstract

**Objective:**

The incidence of non-virus-related hepatocellular carcinoma (NV-HCC) in hepatocellular carcinoma (HCC) is steadily increasing. The aim of this study was to establish a prognostic model to evaluate the overall survival (OS) of NV-HCC patients.

**Methods:**

Overall, 261 patients with NV-HCC were enrolled in this study. A prognostic model was developed by using LASSO-Cox regression analysis. The prognostic power was appraised by the concordance index (C-index), and the time-dependent receiver operating characteristic curve (TD-ROC). Kaplan–Meier (K–M) survival analysis was used to evaluate the predictive ability in the respective subgroups stratified by the prognostic model risk score. A nomogram for survival prediction was established by integrating the prognostic model, TNM stage, and treatment.

**Results:**

According to the LASSO-Cox regression results, the number of nodules, lymphocyte-to-monocyte ratio (LMR), prognostic nutritional index (PNI), alkaline phosphatase (ALP), aspartate aminotransferase (AST)/alanine aminotransferase (ALT) ratio (SLR) and C-reactive protein (CRP) were included for prognostic model construction. The C-index of the prognostic model was 0.759 (95% CI 0.723–0.797) in the development cohort and 0.796 (95% CI 0.737–0.855) in the validation cohort, and its predictive ability was better than TNM stage and treatment. The TD-ROC showed similar results. K–M survival analysis showed that NV-HCC patients with low risk scores had a better prognosis (*P* < 0.05). A nomogram based on the prognostic model, TNM stage, and treatment was constructed with sufficient discriminatory power with C-indexes of 0.78 and 0.85 in the development and validation cohort, respectively.

**Conclusion:**

For NV-HCC, this prognostic model could predict an OS benefit for patients, which may assist clinicians in designing individualized therapeutic strategies.

## Introduction

Hepatocellular carcinoma (HCC) is one of the most dominant malignant tumours in the world, and there are more than 840,000 new cases and over 780,000 deaths per year [1, 2]. Risk factors for HCC include hepatitis B virus (HBV) and hepatitis C virus (HCV), alcohol addiction, nonalcoholic fatty liver disease, obesity, diabetes mellitus, moldy food containing aflatoxin and so on [3]. Patients infected with HBV or HCV accounted for over 80% of HCC cases [4]. However, changing lifestyles and increasing HBV vaccination rates and more efficacious antiviral treatments have changed the global epidemiology of HCC [5]. The incidence of non-virus-related HCC (NV-HCC) is increasing due to fatty liver disease, obesity and insulin resistance [6]. Studies have shown that HBV deoxyribonucleic acid (DNA) may lead to worse liver function and more complications [7]. Thus, HCC patients with HBV have lower overall survival (OS) and disease-free survival (DFS) than NV-HCC patients [8, 9]. These results indicate that NV-HCC and hepatitis B-positive HCC have different clinicopathological features, prognostic factors and clinical outcomes. Thus, a distinct prognostic model for NV-HCC is needed. Recently, numerous prognostic survival models have been established for HCC patients [10, 11]. However, there are few reports on prognostic models for NV-HCC. Therefore, to facilitate clinical counseling and the individualized prediction of survival for NV-HCC, it is necessary to construct a new prognostic model to assess the specific prognosis of NV-HCC.

Numerous studies have reported that clinical characteristics and routine laboratory examinations of blood are prognostic predictors for HCC, including tumor size, HBV DNA [12], alpha-fetoprotein (AFP) [13], neutrophil/lymphocyte ratio (NLR) [14], and alkaline phosphatase (ALP) [15]. Increasing AFP levels were associated with worse survival and higher recurrence rates in patients with HCC [16]. Witjes et al. reported that high AST levels were linked to worse OS in patients with HCC [17]. The NLR is a prognostic factor affecting survival and recurrence in living-donor liver transplantation for HCC [18]. Based on ALP, tumor size, liver cirrhosis, microvascular invasion, and other factors, a nomogram was established to evaluate the prognosis of HCC [12]. However, it is a challenge to screen and combine multiple factors into a prognostic system for NV-HCC.

## Materials and methods

### Patients and laboratory analysis

HCC patients at Sun Yat-sen University Cancer Center from January 2013 to December 2016 were retrospectively reviewed. The inclusion criteria were as follows: (a) patients were not infected with HBV and HCV; (b) patients were diagnosed with HCC by pathology for the first time; (c) patients who had not taken antitumor therapies and anti-inflammatory medicines; (d) patients without a second malignancy in addition to HCC.

The clinical data collected included the following: gender, age, smoking, alcohol drinking history, body mass index (BMI), TNM stage, number of nodules, treatment methods, complete blood count [haemoglobin (HGB), lymphocyte, monocyte, neutrophil, platelets (PLT), red blood cell (RBC), and white blood cell (WBC) counts, neutrophil-to-lymphocyte ratio (NLR), platelet-to-lymphocyte ratio (PLR), lymphocyte-to-monocyte ratio (LMR), prognostic nutritional index (PNI, PNI = serum ALB value (g/L) + 5 × total number of peripheral blood lymphocytes (× 10^9^/L)], liver biochemical tests [total protein (TP), albumin (ALB), alkaline phosphatase (ALP), alanine aminotransferase (ALT), apolipoprotein B (APOB), apolipoprotein A (APOA), aspartate aminotransferase (AST), AST-to-ALT ratio (SLR), cholesterol (CHO), creatinine (CRE), C-reactive protein (CRP), cystatin C (CYSC), high-density lipoprotein (HDL), lactate dehydrogenase (LDH), and low-density lipoprotein (LDL)], coagulation function laboratory tests [activated partial thromboplastin time (APTT), fibrinogen (Fbg), prothrombin time (PT), and thrombin time (TT)], AFP, carbohydrate antigen 19-9 (CA19-9), and carcinoembryonic antigen (CEA). The AI was calculated by the following formula: (TC − HDL-C)/HDL-C [19].

### Statistical analysis

The LASSO-Cox regression model (“glmnet” R package) was utilised to narrow down the candidate indexes and to develop the prognostic model. The prognostic model was calculated after centralisation and standardisation (applying the “scale” function in R) of the development cohort data. The prognostic model formula was as follows: ${\text{Risk score}} = \sum\nolimits_{i}^{{\text{n}}} {Xi \times Yi}$ (n: number of the inclusion index, X: coefficients, Y: survival-related index). The prognostic power of the prognostic risk score, TNM stage, and treatment was appraised by the concordance index (C-index), and the time-dependent receiver operating characteristic curve (TD-ROC). NV-HCC patients were divided into low-risk and high-risk groups based on the risk score’s optimal cut-off (“survminer” R package). The Kaplan–Meier method and log-rank test were used to compare the OS of two risk groups. A box plot based on the prognostic index signatures were generated to show the difference in each index between the high-risk group and the low-risk group. Sankey diagrams were generated to show the patients’ transfers among the prognostic risk score, TNM stage, treatment and survival status. A nomogram was generated with the “nomogram” function in the “nomogram” R package to predict the 1-year, 3-year, and 5-year survival rates of NV-HCC patients. The nomogram for the 1-year, 3-year, and 5-year survival rates were calibrated using calibration curves after comparing the actual survival rate with the predicted probability of survival. The differences of prognostic index signatures between the low-risk and high-risk groups were analysed by the Wilcoxon signed-rank test. All statistical analyses were performed with R software (v3.6.2) and SPSS 25.0 software. A *P* value less than 0.05 was considered statistically significant.

## Results

### Characteristics of the patients

A total of 7511 HCC samples at Sun Yat-sen University Cancer Center from January 2013 to December 2016 were initially screened, and 7250 patients with hepatitis virus infection were excluded. Overall, 261 patients were randomly divided into a development cohort (n = 183) and a validation cohort (n = 78). The clinicopathological variables of NV-HCC patients are described in Table 1. In the development cohort, there were 136 (74.32%) males and 47 (25.68%) females. The mean age of the patients was 61.55 years. In the validation cohort, 57 (73.08%) were males and 21 (26.92%) were females. The mean age of the patients was 62.71 years. The 1-, 3-, and 5-year OS rates for the development and validation cohorts were 68.85%, 49.73%, and 31.15% and 79.49%, 56.41%, and 41.03%, respectively.

**Table 1:** Demographics and clinical characteristics of patients in the development and validation cohort

Characteristic	Development cohort	Validation cohort
	n = (183)	n = (78)
	No. (%) or Mean ± sd	No. (%) or Mean ± sd
Gender		
Male	136 (74.32%)	57 (73.08%)
Female	47 (25.68%)	21 (26.92%)
Age (years)	61.55 ± 12.20	62.71 ± 10.32
Smoking		
Yes	114 (62.30%)	50 (64.10%)
No	69 (37.70%)	28 (35.90%)
Alcohol drink		
Yes	57 (31.15%)	21 (26.92%)
No	126 (68.85%)	57 (73.08%)
BMI (kg/m^2^)		
< 18.00	8 (4.37%)	6 (7.69%)
18.00–24.00	84 (45.90%)	30 (38.46%)
> 24.00	91 (49.73%)	42 (53.85%)
Number of nodules		
≤ 3	117(63.93%)	52(66.67%)
> 3	66(36.07%)	26(33.33%)
TNM stage^a^		
I	44 (24.04%)	28 (35.90%)
II	33 (18.03%)	13 (16.67%)
III	48 (26.23%)	25 (32.05%)
IV	58 (31.70%)	12 (15.38%)
Treatment		
Surgery	99 (54.10%)	49 (62.82%)
Chemotherapy	84 (45.90%)	29 (37.18%)
WBC (10^9^/L)	7.78 ± 2.96	7.59 ± 2.77
Neutrophils (10^9^/L)	5.06 ± 2.65	4.97 ± 2.48
Lymphocyte (10^9^/L)	1.80 ± 0.73	1.83 ± 0.61
Monocyte (10 ^9^/L)	0.93 ± 3.00	0.57 ± 0.25
Platelet (10 ^9^/L)	246.01 ± 102.92	227.88 ± 86.42
HGB (g/L)	134.19 ± 21.81	136.37 ± 17.88
NLR	3.28 ± 2.70	3.01 ± 1.63
RBC (10 ^9^/L)	4.58 ± 0.70	4.57 ± 0.63
LMR	3.29 ± 1.57	3.66 ± 1.65
PLR	153.56 ± 84.48	137.95 ± 70.84
APTT (s)	28.12 ± 5.00	27.7 ± 4.16
Fbg (g/L)	3.71 ± 1.29	3.35 ± 0.97
PT (s)	11.98 ± 1.28	12.98 ± 12.53
TT (s)	18.63 ± 1.53	18.62 ± 1.55
TP (g/L)	73.21 ± 6.95	73.42 ± 5.48
ALB (g/L)	40.60 ± 5.45	42.42 ± 4.21
ALP (U/L)	152.07 ± 127.84	154.43 ± 245.92
ALT (U/L)	59.30 ± 198.73	48.51 ± 59.70
AST (U/L)	68.71 ± 162.13	58.86 ± 97.39
SLR	1.48 ± 0.94	1.36 ± 1.01
CRE (μmol/L)	72.85 ± 20.27	71.16 ± 19.11
CRP (mg/L)	24.58 ± 41.53	17.38 ± 32.88
CHO (mmol/L)	5.23 ± 2.38	5.87 ± 8.84
APOA (g/L)	1.16 ± 0.35	1.42 ± 1.75
APOB (g/L)	1.03 ± 0.38	0.97 ± 0.28
ABR	1.28 ± 0.60	1.64 ± 2.34
LDH (U/L)	263.24 ± 224.22	250.79 ± 209.46
LDL (mmol/L)	3.35 ± 1.79	3.13 ± 1.19
HDL (U/L)	1.16 ± 0.43	1.20 ± 0.32
AI (g/L)	4.01 ± 2.99	3.87 ± 5.66
LHR	3.20 ± 1.97	2.72 ± 1.08
Cys-C (mg/L)	1.03 ± 0.22	0.99 ± 0.24
CEA (ng/mL)	117.03 ± 1364.80	13.56 ± 65.19
AFP (ng/mL)	11,851.07 ± 31,860.00	8482.72 ± 28,890.22
CA199 (U/mL)	201.12 ± 1507.19	411.82 ± 2134.85
PNI	49.61 ± 7.37	51.59 ± 5.94

*BMI* body mass index, *TNM* Tumor Node Metastasis stage, *Rad* radiotherapy, *Che* chemotherapy, *WBC* white blood cell, *HGB* hemoglobin, *NLR* neutrophil/lymphocyte ratio, *RBC* red blood cell count, *LMR* lymphocyte/monocyte ratio, *PLR* platelet/lymphocyte ratio, *APTT* activated partial thromboplastin time, *Fbg* fibrinogen, *PT* prothrombin time, *TT* thrombin time, *TP* total protein, *ALB* albumin, *ALP* alkaline phosphatase, *ALT* alanine aminotransferase, *AST* aspartate aminotransferase, *SLR* AST/ALT ratio, *CRE* creatinine, *CRP* C-reactive protein, *CHO* cholesterol, *APOA* apolipoprotein AI, *APOB* apolipoprotein B, *ABR* APOA/APOB ratio, *LDH* lactic dehydrogenase, *LDL* low density lipoprotein, *HDL* high density lipoprotein, *AI* (TC − HDL-C)/HDL-C, *LHR* LDL/HDL ratio, *Cys-C* cystatin C, *CEA* carcinoembryonic antigen, *AFP* alpha-fetoprotein, *CA199* Carbohydrate antigen 199, *PNI* prognostic nutritional index

^a^TNM stage was classified according to the AJCC 8th TNM staging system

### Prognostic model construction and evaluation

**Fig. 1:**
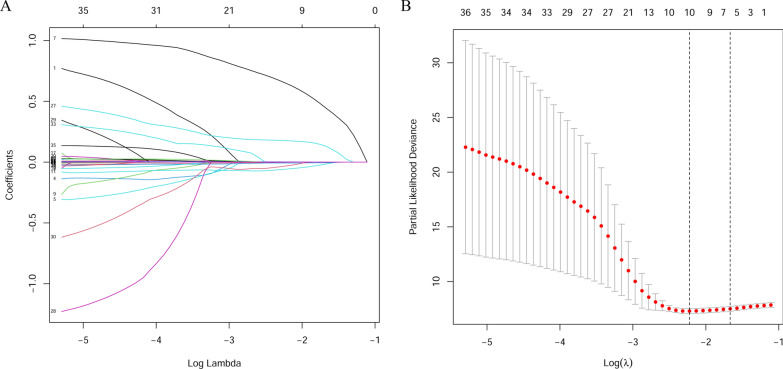
Construction of the prognostic model in the development cohort. LASSO-Cox regression analysis selected potential indicators (**A**). Cross-validation for tuning the parameter selection in the LASSO-Cox regression (**B**)

**Fig. 2:**
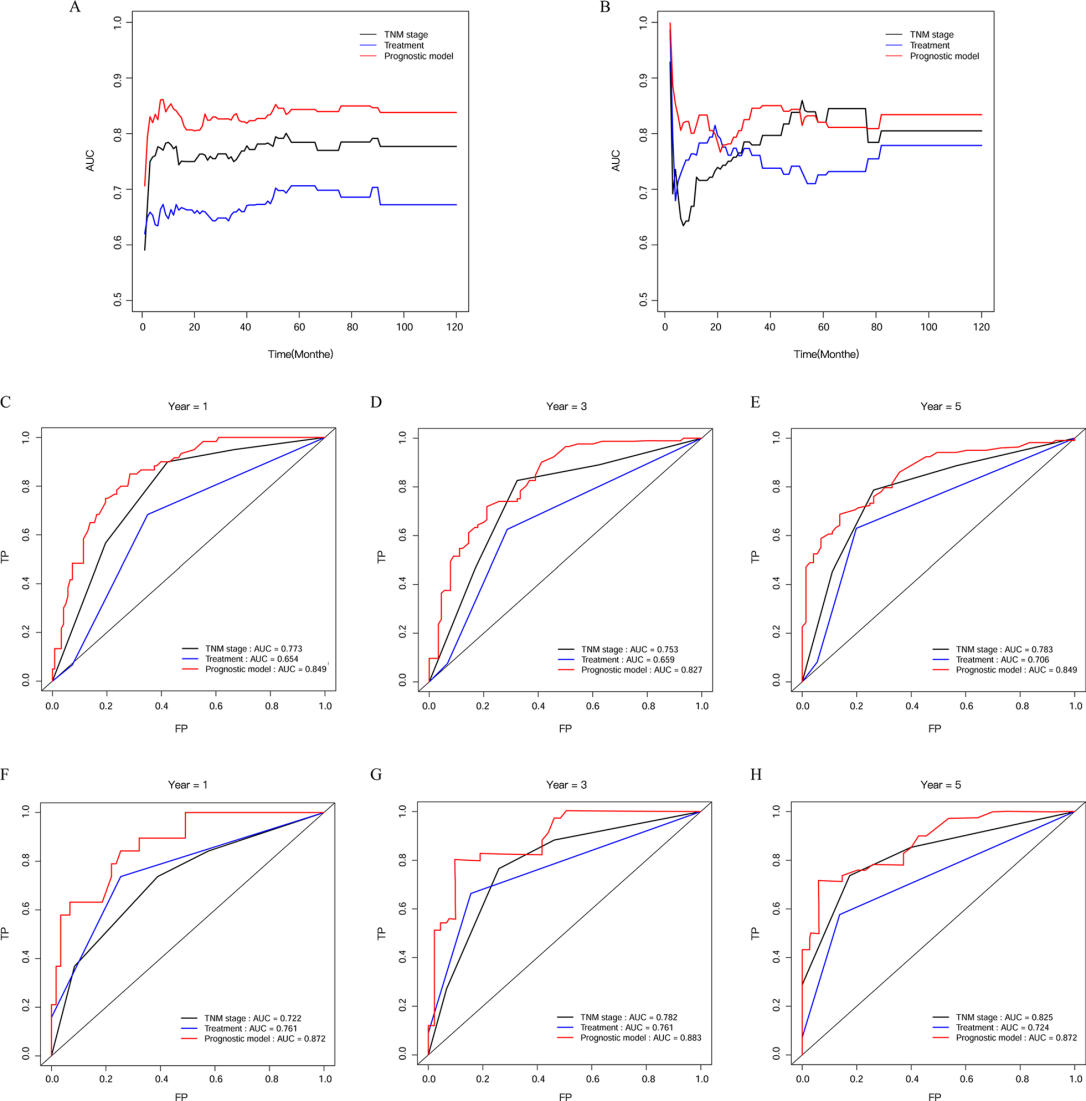
Time-dependent ROC in the development cohort (**A**). Time-dependent ROC in the validation cohort (**B**). ROC curves appraise the predictive efficiency of the prognostic model, TNM stage, and treatment for 1-year OS, 3-year OS, and 5-year OS in the development (**C**–**E**) and validation cohort (**F**–**H**)

In the primary cohort, a 6-prognostic index (number of nodules, LMR, PNI, ALP, SLR and CRP) signature was constructed by performing LASSO-Cox regression analysis (Fig. 1A, B). The prognostic model was calculated as follows: Risk score = (0.4419 * number of nodules) + (− 0.0156 * LMR) + (− 0.005 * PNI) + (0.001 * ALP) + (0.1301 * SLR) + (0.0001 * CRP). The C-index was used to compare the predictive power of the prognostic model with that of TNM stage and treatment. In the development cohort, the prognostic model achieved a C-index of 0.759 (95% CI 0.723–0.797), which was higher than the C-index of TNM stage (0.708; 95% CI 0.663–0.753; *P* = 0.021) and treatment (0.630; 95% CI 0.582–0.677; *P* < 0.001). In the validation cohort, the C-index of the prognostic model, TNM stage, and treatment were 0.796 (95% CI 0.737–0.855), 0.721 (95% CI 0.647–0.795), and 0.700 (95% CI 0.630–0.770), respectively (Table 2). TD-ROC analysis was performed to evaluate the accuracy of the prognostic model, TNM stage, and treatment in the development cohort (Fig. 2A) and validation cohort (Fig. 2B). The area under the ROC curve (AUC) values of the prognostic model were higher than those of TNM stage and treatment for all cohorts (Fig. 2). For the 1-year OS, the AUCs of the prognostic model, TNM stage, and treatment were 0.849, 0.773, and 0.654, respectively (Fig. 2C). In addition, for the 3-year OS, and 5-year OS, the prognostic model also had higher AUC values than TNM stage and treatment (Fig. 2D, E).

**Table 2:** The C-index of the prognostic model, TNM stage, and treatment for prediction of NV-HCC OS in the development cohort and validation cohort

Factors	C-index (95% CI)	*P*
For development cohort		
Prognostic model	0.759 (0.723–0.797)	
TNM stage	0.708 (0.663–0.753)	
Treatment	0.630 (0.582–0.677)	
Prognostic model vs TNM stage		0.021
Prognostic model vs treatment		< 0.001
For validation cohort		
Prognostic model	0.796 (0.737–0.855)	
TNM stage	0.721 (0.647–0.795)	
Treatment	0.700 (0.630–0.770)	
Prognostic model vs TNM stage		0.022
Prognostic model vs treatment		0.029

P values are calculated based on normal approximation using function rcorrp.cens in Hmisc package

*C-index* concordance index, *CI* confidence interval

### Risk stratification of OS based on the prognostic model

**Fig. 3:**
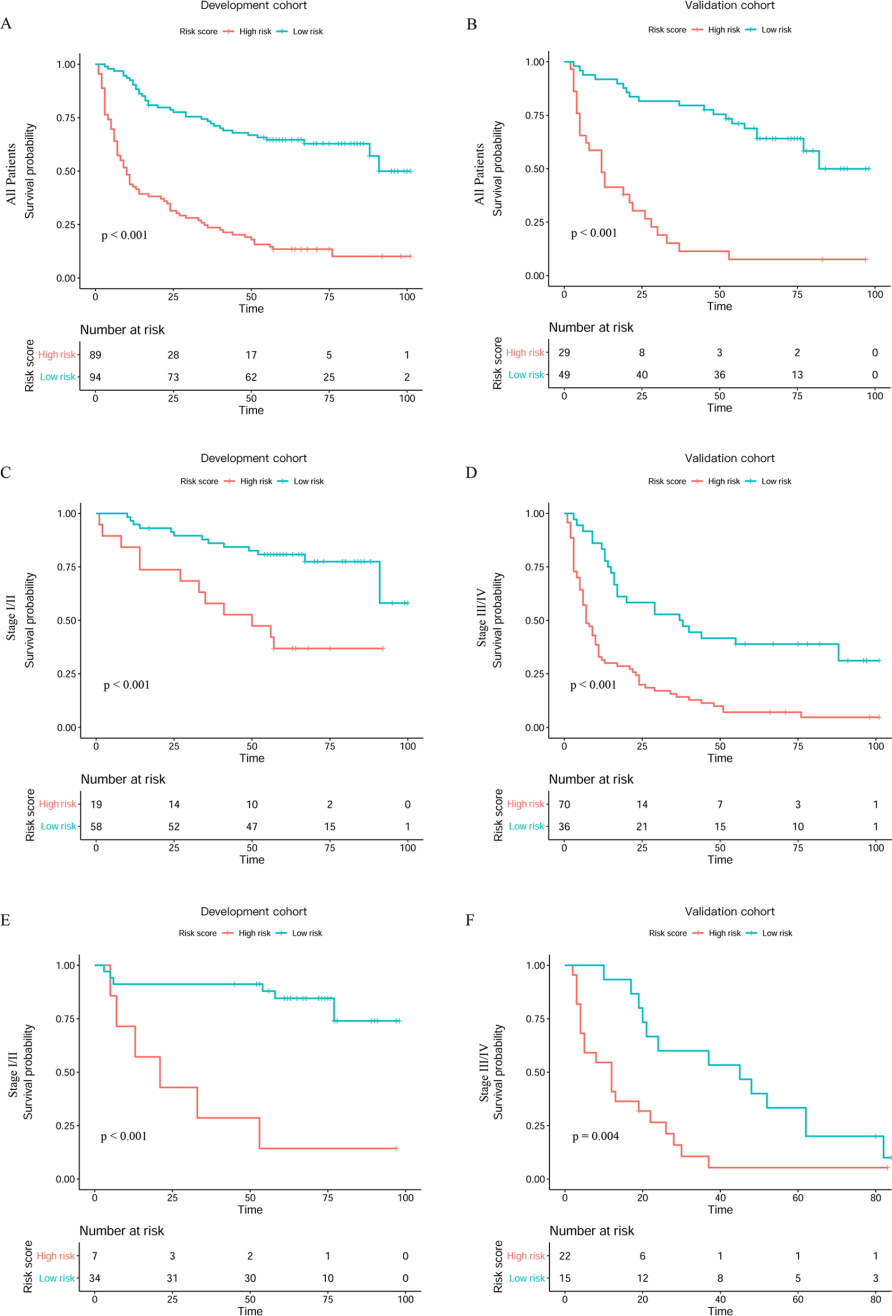
Kaplan–Meier curves for the OS of patients in the high-risk group and low-risk group in the development cohort and the validation cohort: all patients (**A**, **B**); stage I/II (**C**, **E**); stage III/IV (**D**, **F**)

Based on the optimal cut-off of the risk score, all patients were divided into a low-risk group (< 0.59) and a high-risk group (≥ 0.59). A notable difference in OS was detected between the low-risk and high-risk groups, and the high-risk group had shorter OS than the low-risk group in the development cohort (Fig. 3A) and validation cohort (Fig. 3B). Moreover, there was a significant difference between the high-risk and low-risk groups for stage I/II and stage III/IV in the development cohort (Fig. 3C, *P* < 0.001; Fig. 3D, *P* < 0.001) and in the validation cohort (Fig. 3E, *P* < 0.001; Fig. 3F, *P* = 0.004).

**Fig. 4:**
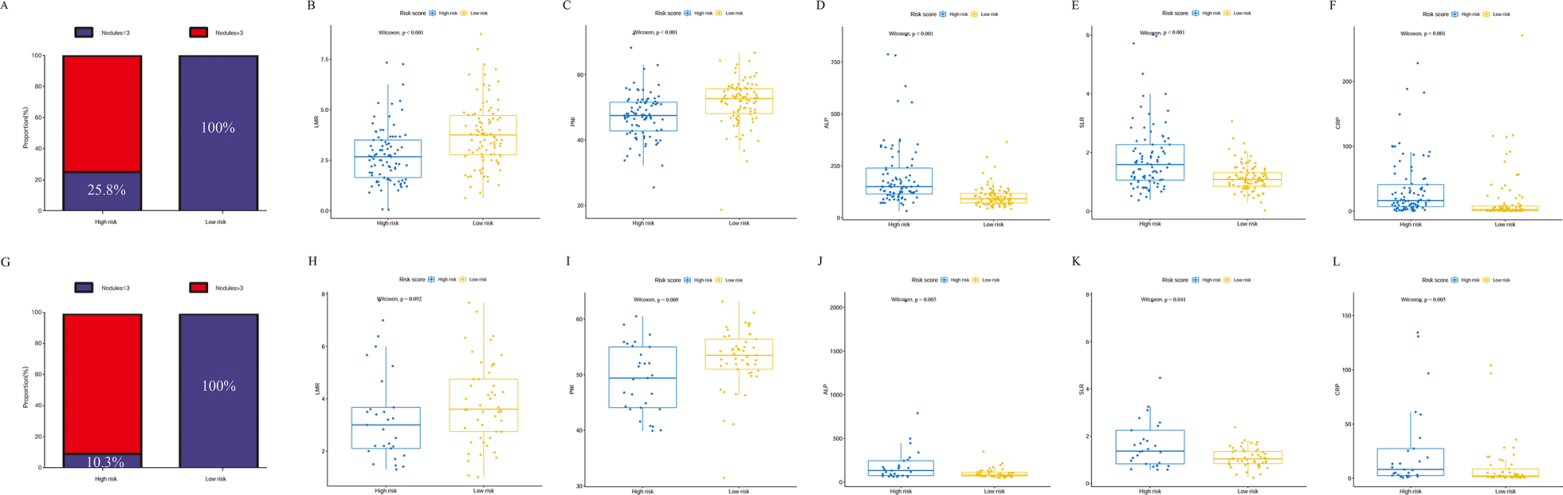
Differences between the high-risk and low-risk group in the number of nodules, LMR, PNI, ALP, SLR and CRP, which were analysed using boxplots in the development (**A**–**F**) and validation cohort (**G**–**L**). Number of nodules (**A**, **G**); LMR (**B**, **H**); PNI (**C**, **I**); ALP (**D**, **J**); SLR (**E**, **K**); CRP (**F**, **L**)

**Fig. 5:**
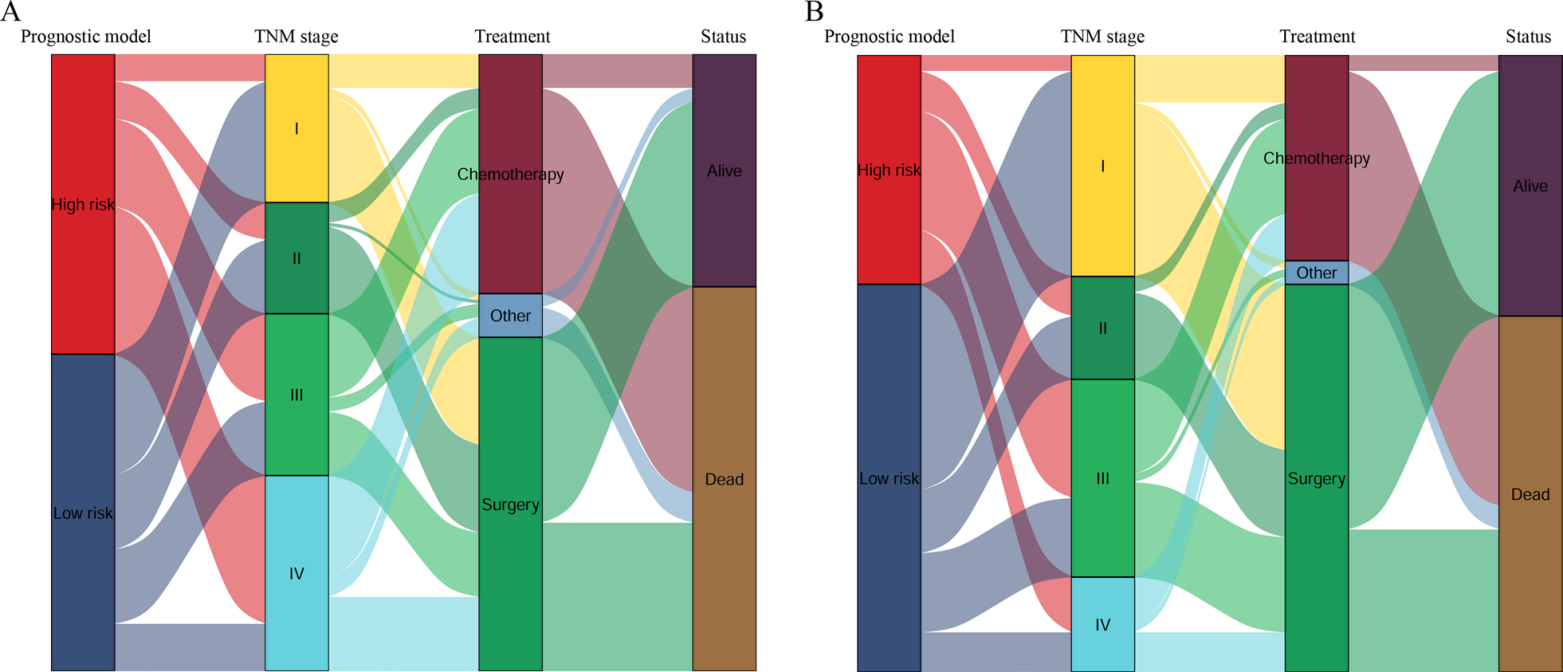
The Sankey diagrams showed the patients’ transfers between the prognostic risk score, TNM stage, treatment and survival status in the development (**A**) and validation cohort (**B**), the thicker line, the more patients

The differences between the high-risk and low-risk groups in the number of nodules, LMR, PNI, ALP, SLR and CRP were analysed using a boxplot (Fig. 4). The number of nodules and ALP, SLR and CRP levels in the high-risk group were significantly higher than those in the low-risk group in the development (*P* < 0.05; Fig. 4A, D–F) and validation cohorts (*P* < 0.05; Fig. 4G, J–L). In the development cohort, LMR and PNI levels in the low-risk group were significantly higher than those in the high-risk group (*P* < 0.05; Fig. 4B, C). However, there was no significant difference between the two groups regarding the LMR levels in the validation cohort (*P* = 0.092, Fig. 4H). In addition, Sankey diagrams showed that most of the low-risk group patients shifted to stage I/II, were treated with surgery, and had a higher level of survival status in the development (Fig. 5A) and validation cohorts (Fig. 5B).

### The nomogram for the prediction of OS

**Fig. 6:**
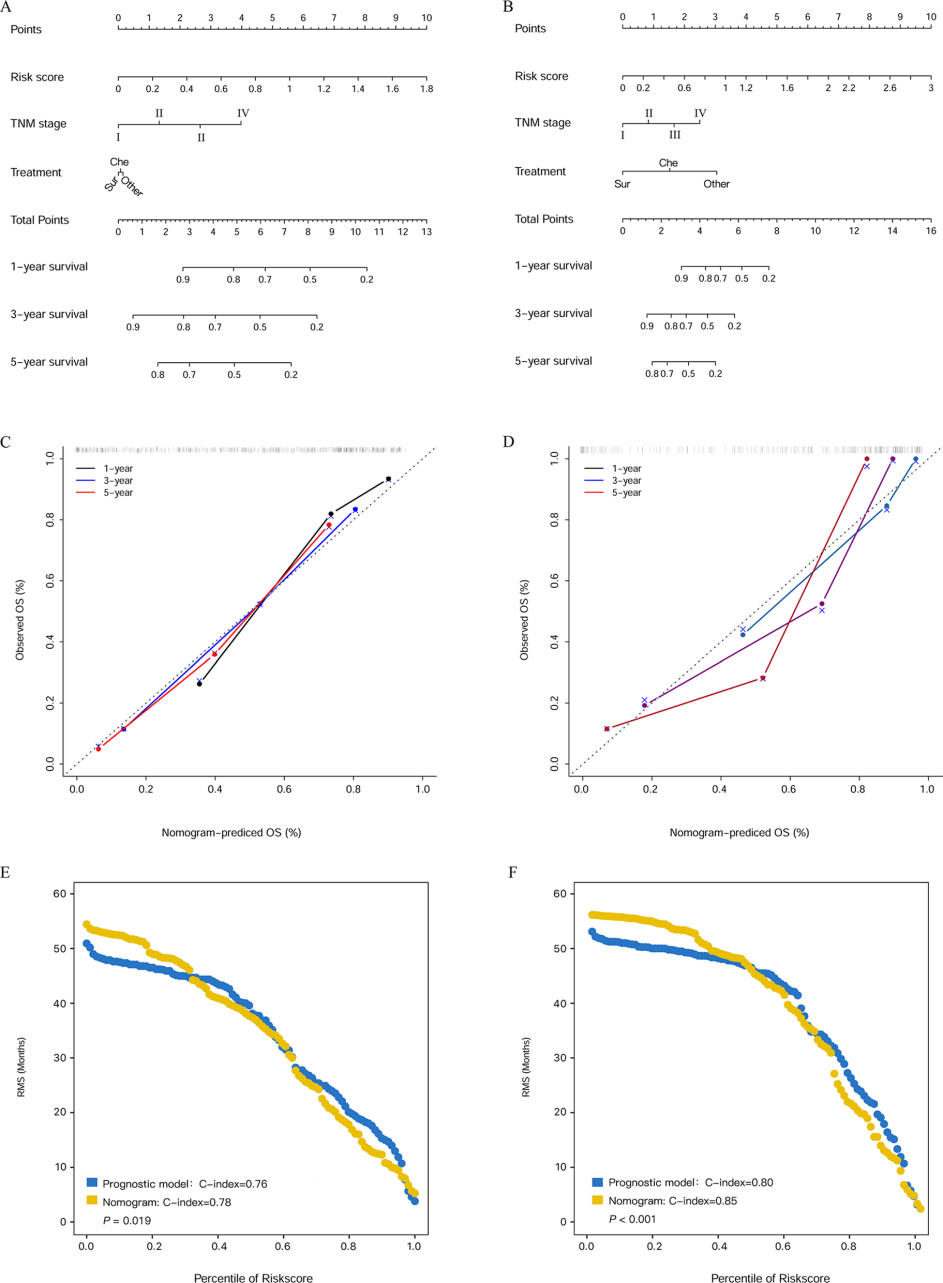
Nomogram for patients with NV-HCC in the development cohort (**A**) and the validation cohort (**B**). Calibration curves of the nomogram for OS in the two cohort (**C**, **D**). Restricted mean survival (RMS) curves for the prognostic model and nomogram in the development cohort (**E**) and the validation cohort (**F**)

Based on the prognostic risk score, TNM stage, and treatment, we created a prognostic nomogram for the prediction of OS in the two cohorts (Fig. 6A, B). In the development (Fig. 6C) and validation cohorts (Fig. 6D), calibration curves of 1-year, 3-year, and 5-year survival showed optimal consistency between the prediction established in the nomogram and actual observations. In the development cohort, the C-index of the prognostic model and nomogram were 0.76 and 0.78, respectively (*P* = 0.019) (Fig. 6E). Similarly, the nomogram model achieved a higher C-index (0.85) than the prognostic model (0.80) (*P* < 0.001) (Fig. 6F).

## Discussion

HCC is a leading cause of cancer related death worldwide [20]. Most HCC patients have a poor prognosis, and the 5-year OS is only 12.1% [21]. HBV and HCV infection are major important risk factors for HCC. Recently, with lifestyle changes and efficient vaccination strategies, the number of HCC virus patients has decreased, and the number of NV-HCC patients is increasing [5]. In Taiwan, due to the universal newborn vaccination program, HCC incidence is significantly lower in younger persons who were vaccinated than in those who were not vaccinated at birth [22]. However, there are few studies predicting the occurrence of NV-HCC. TNM stage is commonly used to predict the prognosis for many cancers [23, 24]. However, studies reported that patients with the same TNM stage had different clinical outcomes [25]. This phenomenon indicates that the TNM stage utilized for guidance of the different treatments is insufficient. Therefore, we developed a prognostic model for NV-HCC. LASSO-Cox regression is a useful tool for feature selection and regularization to improve the accuracy of statistical models [26]. In this study, we constructed a prognostic model for NV-HCC to further guide clinical treatment by using LASSO-Cox regression analysis of the pathological results and clinical laboratory test results.

In this study, by using LASSO-Cox regression analysis, six predictive indicators (number of nodules, LMR, PNI, ALP, SLR and CRP) were selected for the prediction of NV-HCC prognosis. Then, we constructed a prognostic model based on the six factors for NV-HCC patients. The risk score was calculated as follows: Risk score = (0.4419 * number of nodules) + (− 0.0156 * LMR) + (− 0.005 * PNI) + (0.001 * ALP) + (0.1301 * SLR) + (0.0001 * CRP). Based on the risk score, the NV-HCC patients were divided into a low-risk group (risk score < 0.59) and a high-risk group (risk score ≥ 0.59). Kaplan–Meier curves revealed that the high-risk group of NV-HCC patients had a poor OS (*P* < 0.001). The prognostic score model achieved a higher AUC than the TNM stage and treatment for the 1-year OS, 3-year OS, and 5-year OS. Moreover, we constructed a nomogram that can help to predict OS in NV-HCC patients, which integrated the prognostic score, TNM stage and treatment. Notably, according to our study, the nomogram model was a more powerful predictive factor of OS for NV-HCC patients than the prognostic model, and the C-index of the nomogram model (0.78 and 0.85) was higher than the C-index of the prognostic risk score model (0.76 and 0.80) in the development cohort and in the validation cohort. Moreover, in the development cohort and validation cohort, the heatmaps and waterfall plots of the clinical features also indicated that patients who had shorter OS were mainly distributed in the high-risk group, TNM stage III or IV group, and treatment with chemotherapy group.

The prognosis of NV-HCC patients is closely related to the number of nodules in the liver. Mazzotta et al. reported that patients who had more than 5 HCC nodules during the waiting period had a high risk of post liver transplantation recurrence and death [27]. Markers of the inflammatory response, including LMR, SLR, lymphocytes, NLR, and CRP, play important roles in the progression of many cancers [28, 29]. Studies have revealed that LMR is associated with survival in patients with breast cancer, and a low LMR indicates poor prognosis in stage I–III breast cancer [30]. LMR markedly increased the level of tumour-infiltrating Th17 cells and promoted tumour growth in HCC [31]. Serum ALP, AST, ALT, and CRP are biomarkers of systemic inflammation and immune activation, and can be used to evaluate liver function [32]. The elevation of ALP has been demonstrated to predict poor prognosis in esophageal squamous cell carcinoma and pancreatic cancer [33, 34]. Moreover, ALP was incorporated into prognostic models for many cancers, including HCC and gastric cancer [35, 36]. Our previous study showed that the LSR is an independent prognostic factor for gastric cancer [37]. We also established a nomogram based on age, stage status, and SLR, which had a more accurate prognostic prediction for patients with gastric cancer [38]. CRP is an indicator of inflammatory response, which combined with increased cytokines, growth factors, activated stroma, and DNA damage, promotes tumour invasion, migration and metastasis [39]. Currently, a low PNI has been shown to be a significant predictor of poor postoperative outcomes and increased mortality in various malignancies, including colorectal cancer, breast cancer, and pancreatic cancer [40–42]. In this study, we used LASSO-Cox regression analysis to identify that the number of nodules, LMR, PNI, ALP, SLR and CRP levels can be used to predict the prognosis of NV-HCC.

There are some limitations in this study that should be noted. First, this model employed data from one medical centre. Multicentre data are needed to further verify the performance of the model. Second, this study has small sample size of NV-HCC patients in the development and validation cohorts. Therefore, a larger cohort is urgently needed to further verify the model of our study. In addition, this study only analysed the OS of NV-HCC patients, and it is uncertain whether DFS and progression-free survival (PFS) can be verified.

## Conclusion

In summary, we established a prognostic model for NV-HCC based on 6 factors (number of nodules, LMR, PNI, ALP, SLR and CRP) via LASSO-Cox analysis, and found that it can be used to predict OS in NV-HCC patients. Moreover, a nomogram was constructed that integrated the prognostic model, TNM stage, and treatment. The prognostic model can provide a more precise estimation for patients with NV-HCC.
